# OCT Study of Mechanical Properties Associated with Trabecular Meshwork and Collector Channel Motion in Human Eyes

**DOI:** 10.1371/journal.pone.0162048

**Published:** 2016-09-06

**Authors:** Chen Xin, Murray Johnstone, Ningli Wang, Ruikang K. Wang

**Affiliations:** 1 Departments of Bioengineering, University of Washington, Seattle, Washington, 98195, United States of America; 2 Department of Ophthalmology, University of Washington, Seattle, Washington, 98104, United States of America; 3 Beijing TongRen Eye Center, Beijing TongRen Hospital, Capital Medical University, Beijing, 100730, China; 4 Department of Ophthalmology, Beijing AnZhen Hospital, Capital Medical University, Beijing, 100029, China; Duke University, UNITED STATES

## Abstract

We report the use of a high-resolution optical coherence tomography (OCT) imaging platform to identify and quantify pressure-dependent aqueous outflow system (AOS) tissue relationships and to infer mechanical stiffness through examination of tissue properties in *ex vivo* human eyes. Five enucleated human eyes are included in this study, with each eye prepared with four equal-sized quadrants, each encompassing 90 degrees of the limbal circumference. In radial limbal segments perfusion pressure within Schlemm’s canal (SC) is controlled by means of a perfusion cannula inserted into the canal lumen, while the other end of the cannula leads to a reservoir at a height that can control the pressure in the cannula. The OCT system images the sample with a spatial resolution of about 5 μm from the trabecular meshwork (TM) surface. Geometric parameters are quantified from the 2D OCT images acquired from the sample subjected to controlled changes in perfusion pressures; parameters include area and height of the lumen of SC, collector channel entrances (CCE) and intrascleral collector channels (ISCC). We show that 3D OCT imaging permits the identification of 3-D relationships of the SC, CCE and ISCC lumen dimensions. Collagen flaps or leaflets are found at CCE that are attached or hinged at only one end, whilst the flaps are connected to the TM by cylindrical structures spanning SC. Increasing static SC pressures resulted in SC lumen enlargement with corresponding enlargement of the CCE and ISCC lumen. Pressure-dependent SC lumen area and height changes are significant at the 0.01 levels for ANOVA, and at the 0.05 for both polynomial curves and Tukey paired comparisons. Dynamic measurements demonstrate a synchronous increase in SC, CCE and ISCC lumen height in response to pressure changes from 0 to 10, 30 or 50 mm Hg, respectively, and the response time is within the 50-millisecond range. From the measured SC volume and corresponding IOP values, we demonstrate that an elastance curve can be developed to infer the mechanical stiffness of the TM by means of quantifying pressure-dependent SC volume changes over a 2 mm radial region of SC. Our study finds pressure-dependent motion of the TM that corresponds to collagen leaflet configuration motion at CCE; the synchronous tissue motion also corresponds with synchrony of SC and CCE lumen dimension changes.

## Introduction

Glaucoma is a leading cause of blindness and intraocular pressure (IOP) is currently the only treatable risk factor [1]. Patients continue to go blind from glaucoma [2] and IOP is the major component associated with a rapid progression rate [3]. Aqueous flow regulation fails in glaucoma resulting in IOP elevation. Clinical evidence indicates that AOS tissue motion is a normal feature of aqueous outflow and is important to IOP regulation [1–7]. Experimental evidence also indicates pressure-dependent AOS tissue motion is normal [8–12].

Regulation of AOS motion is thought to decrease in glaucoma as evidenced by the reduced ability of SC lumen to change dimensions and by diminished pulsatile aqueous outflow in aqueous veins; tissue stiffening is considered to be an underlying cause of both phenomena [1–7]. Elastic modulus determination in normal and glaucoma conditions [13] as well as Wnt and steroid studies implicate increased TM tissue stiffness as a factor in the outflow regulation failure in open angle glaucoma [14,15]. Mechanical stretching such as occurs with the ocular pulse modulates extracellular matrix (ECM) composition of the trabecular beams and the juxtacanalicular tissues [16, 17]. Composition of the ECM in turn modulates tissue elasticity and compliance, properties necessary for the maintenance of normal function.

Cyclic IOP changes alter conventional aqueous outflow and cellular contractile mechanisms [18]. Pressure-induced mechanical stresses lead to alterations in gene expression [19–21], changes in cytoskeletal networks [22] and alterations in signal transduction [20] consistent with active regulation of both cellular and ECM constituent properties that determine tissue stiffness. Tissue elasticity and compliance also set boundaries on the effects pilocarpine can impose on motion of the aqueous outflow pathways [23].

Optical coherence tomography (OCT) has been used to demonstrate that an acute IOP elevation reduces SC dimensions [24]; reduced dimensions are also present in glaucoma eyes [25,26]. Because AOS tissue motion that alters SC dimensions is likely to have an important role in aqueous outflow regulation, the ability to monitor this tissue motion may offer new opportunities to improve glaucoma management and decision-making.

New high-resolution OCT techniques offer advantages of non-invasive, high-speed imaging of tissue structures *in vivo* in real time with <10-μm resolution [27,28]. The techniques are shedding light on dynamic pressure-dependent AOS changes; both animal [23,29,30] and human eyes [25,31–36] are currently being studied with these new modalities. However, even with recently developed OCT systems, the delineation of SC configuration, the characterization of microstructures crossing SC and the detailed relationship between SC and CC remain difficult. The persistent technical limitation is primarily due to the highly scattering nature of relatively thick overlaying sclera tissue. The limitation continues to be a barrier to for visualizing the AOS structures in sufficient detail [26].

We recently developed a high resolution OCT platform that is particularly well suited for visualizing and quantifying the AOS tissue components in excised eyes [29]. The OCT system operates at a 1310 nm central wavelength, providing a spatial resolution of ~5 μm. The resolution is sufficient to delineate important microstructural details of TM, SC and CCE previously unattainable with OCT. To avoid the limitation caused by the highly scattering nature of the overlaying sclera tissue, the OCT platform was designed to expose the TM surface of the AOS of *ex vivo* eyes directly to the imaging system.

Our imaging platform is not only capable of imaging the AOS from the TM surface, but also has an ability to control SC lumen pressure. With the combination of techniques, we have been able to delineate tissue components within the AOS in detail [8,37,38]. This system capability enabled us to explore complex pressure-dependent relationships involving the TM, structures within SC as well as collector channel ostia in *ex-vivo* non-human primate (NHP) eyes [29].

Findings in NHP eyes suggest that the linked changes in configuration of the TM and hinged collagen flaps that control CCE dimensions may have a role in the control of aqueous flow. Factors distal to SC that may contribute to the control of flow represent an important consideration in both normal and glaucoma eyes. However, correlations between pressure-dependent AOS relationships involving the TM, SC and CCE have not previously been explored in human eyes.

The goal of the current study is to image and quantify the pressure-dependent tissue relationships within the AOS in *ex vivo* human eyes by employing our recently developed high-resolution OCT platform. We examine pressure-dependent tissue responses of the TM, SC and CCE. In addition, we explore the synchronized linkage between the motion of the TM and the changes in CCE lumen dimensions. Lastly, we describe a method to explore TM elastance properties.

## Methods

### System Setup

**Fig 1:**
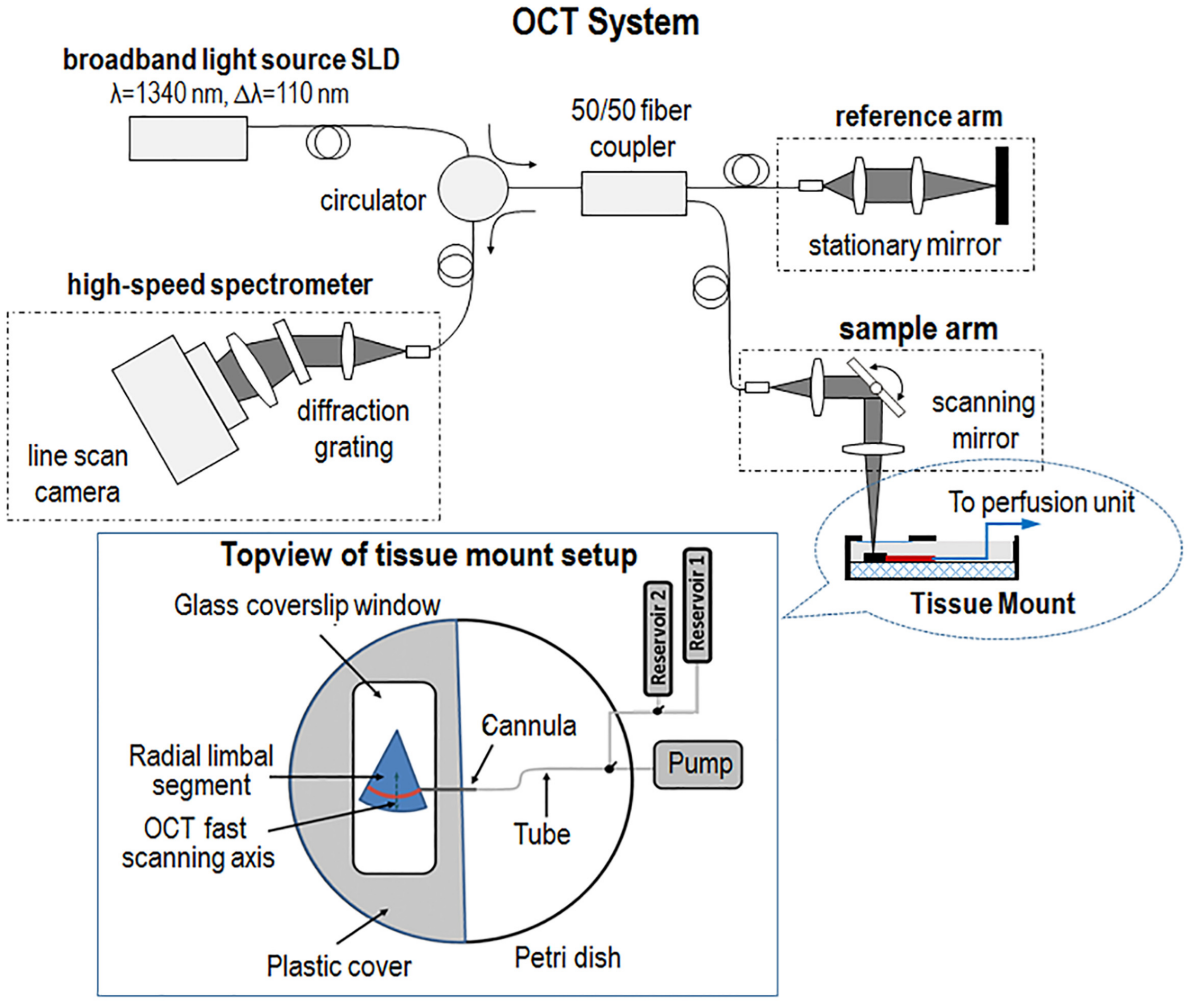
Schematic diagram of the experimental setup, including an SD-OCT system and a perfusion unit. The perfusion unit can be used to induce pressure transients. A single reservoir can control static pressures in the cannula inserted into Schlemm’s canal (SC), while switching between two reservoirs provides a means of examining dynamic changes. A radial limbal segment is placed with the trabecular meshwork facing upward toward the OCT imaging beam and a cannula is inserted into SC.

In this study, we use an experimental OCT system platform that was previously described in detail [29]. Briefly, the platform consists of a perfusion unit and an OCT imaging system. The perfusion unit infuses fluid into SC lumen in radial limbal segments during imaging with the OCT system (Fig 1). The OCT system is a spectral domain configuration (i.e., SD-OCT) powered by a broadband light source with a 110-nm spectral bandwidth centered on 1340 nm. The light source provides an axial resolution of ~ 7.2 μm measured in air (~5 μm in tissue). OCT signal detection is by a high-speed spectrometer equipped with an InGaAs line scan camera with 1024 pixels capable of 92 KHz A-line rate. The objective lens in the sample arm is designed to have a lateral resolution of ~5 μm at the AOS. The measured axial imaging range is ~2.2 mm in tissue, providing a depth of focus of ~0.5 nm, a range sufficient for the purpose of imaging the AOS system in this study. A sample beam power of 2.5 mW provides a measured dynamic range of 105 dB at the focal spot.

The radial limbal segments were pinned to a layer of silicone inside a Petrie dish to maintain a stable tissue position with the TM facing the probe beam of the OCT system. The tissue was continuously covered by Hank’s balanced salt solution. A fluid-glass interface was maintained between the sample and the OCT scanner using a previously described setup [29]. The interface eliminates air-fluid surface motion artifact [39] that proved to be particularly valuable when performing dynamic imaging of the TM.

The cannula was carefully inserted into the SC lumen under direct observation with an optical microscope. Placement and maintenance of the cannula position throughout the experiments was achieved with a micromanipulator. The perfusion reservoirs were connected to the cannula. The perfusion setup permitted switching between two reservoirs at different heights [reservoir 1 and reservoir 2 in Fig 1]. Experimentally controlled static pressures were achieved by attachment to a single reservoir while dynamic pressure changes were achieved by means of rapidly switching a three-way valve between reservoirs at different heights.

### Tissue Preparation and Data Acquisition

Five human eyes without known ocular disease were obtained from Sightlife^™^ within 24 h after death. The mean age of the donors was 67.4 ± 10.1 years, ranging from 53 to 79 [4 female and 1 male; Race: Caucasian 4, Other 1]. Four equal-sized quadrants each encompassing 90 degrees of the limbal circumference were prepared from each sample.

**Fig 2:**
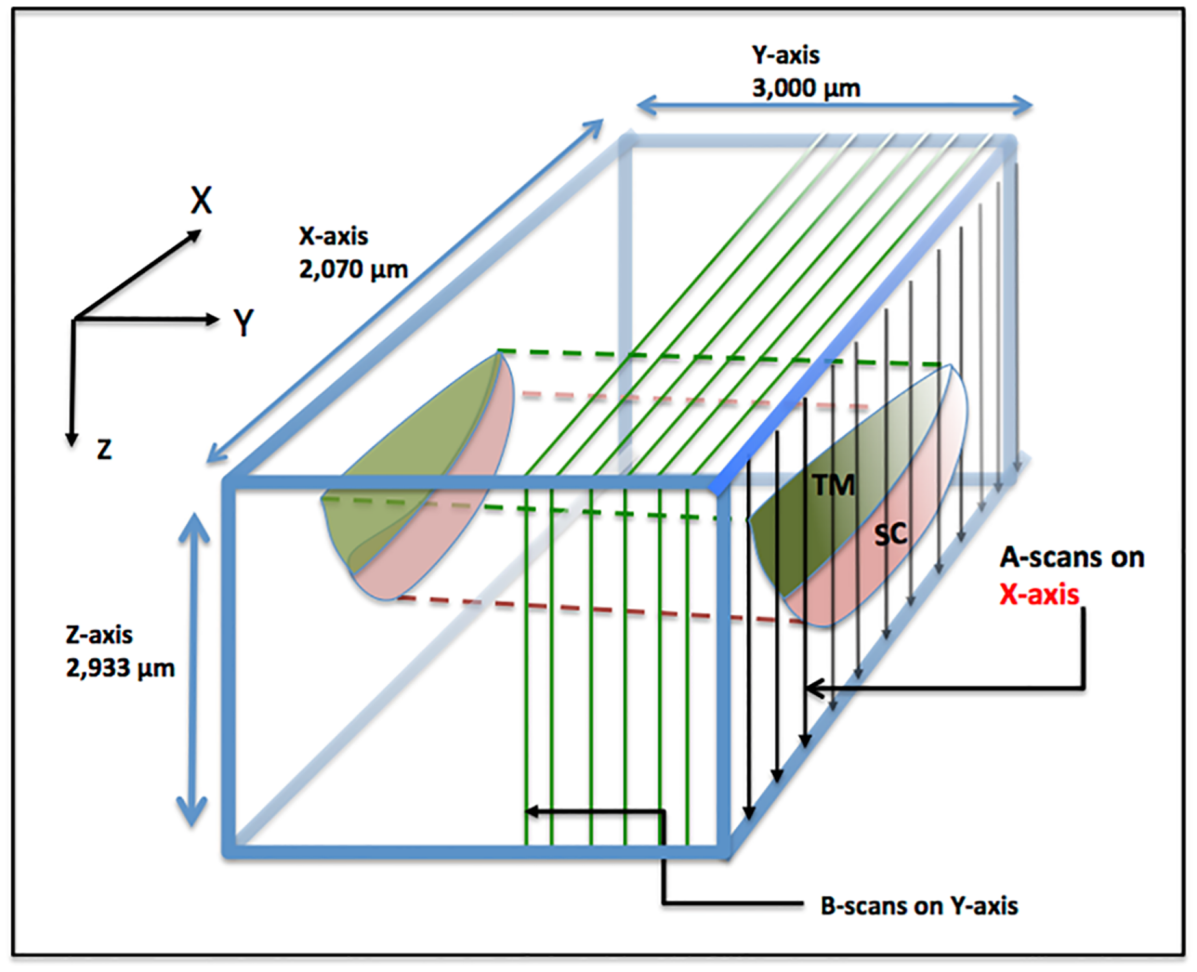
Schematic illustration of the OCT scanning protocol. B-scans are performed as a parallel series of sequential cross sections along the Y-axis, which represents the circumferential direction along Schlemm’s canal. A-scans are performed sequentially along the X-axis in the plane of the B-scan. The A-scans traverse along the Z-axis from the trabecular meshwork surface to the surface of the sclera with each A-scan oriented orthogonal to the X-axis. Scanning dimensions in each axis are as indicated.

Structural relationships of the TM, SC and CCE region were evaluated from the images captured by the SD-OCT as described above. The SD-OCT has two modes of imaging, i.e., 3-D and MB-scan imaging modes. Fig 2 depicts the overall scanning configuration. The 3-D scanning design provides a scanning area of 2 mm × 3 mm at the regions of interest. Each 3-D image is composed of 512 equally-space B-scans in the y-axis (Fig 2) with each B-scan having 360 equally spaced A-lines in the x-axis. The imaging speed of the OCT system is 200 B-scans/second.

The MB-mode imaging design provides repeated B-scans over time at one 2D cross-section of the sample to determine pressure-dependent tissue responses. The duration of the MB-mode scan is 7 seconds, covering the intervals before, during and after the change in pressure determined by switching the 3-way valve between reservoirs.

To determine the geometrical properties of SC, the OCT images obtained at steady state pressures use a semi-automatic segmentation algorithm [40] to isolate SC and CC permitting automated generation of an elastance curve to avoid subjective interpretation. After segmentation, the 3-D volumetric images of SC are assembled from the 2-D cross-sectional images of the canal, permitting analysis of SC area, height and changes in height.

A one-way analysis of variance (ANOVA) was used to determine differences among groups. Tukey paired comparisons were used to determine the variance differences between each two groups. Multiple polynomial regression analysis was also performed to evaluate the effect of pressure on the outflow tissue configuration. Statistical analysis was done by Origin (version 8.5) and SPSS (version 17.0) software. To determine how well the data fit a best-fit polynomial regression line, *r*^*2*^ and *p*-values were determined. A *p* value < 0.01 was considered statistically significant for ANOVA and *r*^*2*^. A p< 0.05 was considered significant for Turkey paired comparisons.

## Results

**Fig 3:**
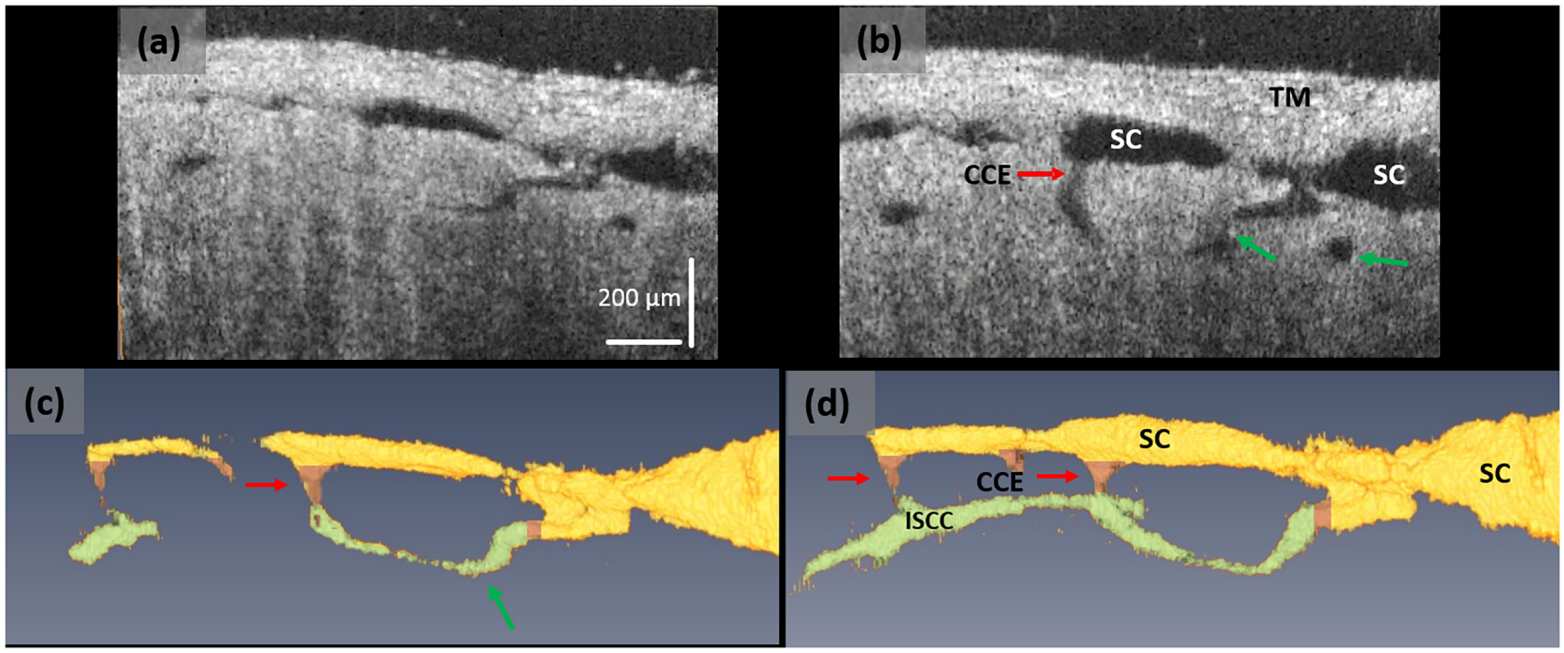
The SD-OCT system provides detailed images of microstructures within the AOS system. (**a**) A representative OCT cross-sectional image cut longitudinally through SC at a pressure of 10 mmHg, and (**b**) the corresponding cross-section at 50 mm Hg. Images (**c**) and (**d**) are the 3D reconstructions of SC, CCE and ISCC within the same specimen under the pressures of (**a**) 10 and (**b**) 50 mm Hg, respectively. With the increase of pressure, the ISCCs move out of the 2D plane and are oriented closer to the observer in the 3D sections. Yellow color identifies the region of SC, pink the CCE, and green the ISCC. In image (**c)** at 10 mm Hg pressure, SC and the ISCC are smaller and in some areas absent in comparison with image (**d**) at 10 mm Hg.

OCT imaging permitted identification of SC, CCE, intrascleral collector channels (ISCC) and their 3D relationships. Fig 3 shows an example of an acquired 3D OCT image using perfusion pressures of 10 and 50 mm Hg. The figure demonstrates an ISCC oriented circumferentially adjacent to SC; a similar configuration is described with AOS casting studies of the deep scleral plexus [41,42]. The TM lamellae are visible with a horizontally oriented pattern distinctly different from the more homogenous appearance of the sclera.

The dimensions of the lumen of SC, the lumen of the CC and its entrance (CCE) identified by red arrow is barely visible at 10 mmHg (Fig 3a); each becomes enlarged at 50 mm Hg (Fig 3b, red arrow). The ISCC dimensions also increase (green arrows). Some regions of SC and ISCC are even absent at low pressures but become visible at the higher pressure. The altered anatomic configuration associated with changes in static pressures provides evidence that both SC and ISCC undergo pressure-dependent changes in shape.

**Fig 4:**
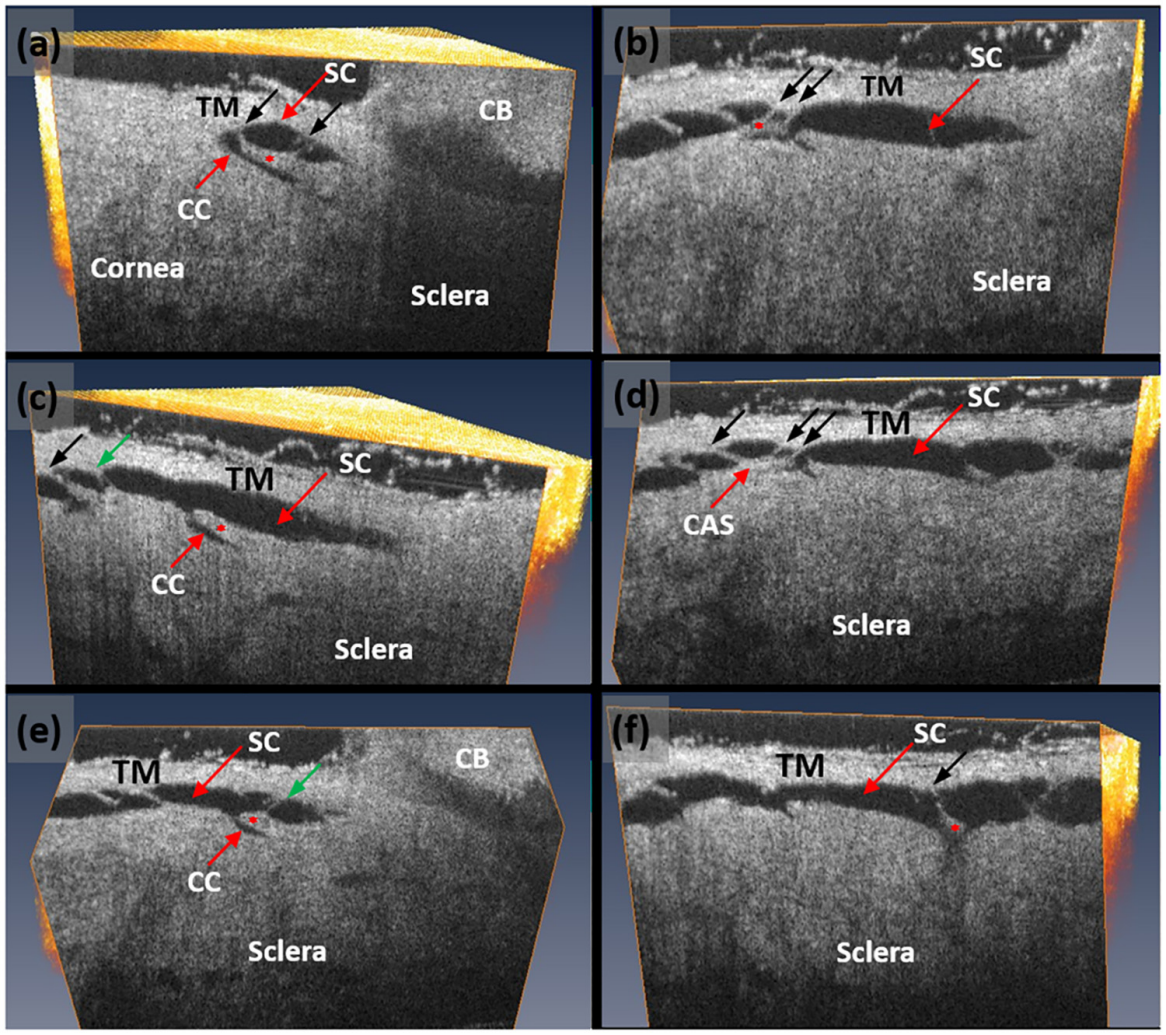
High-resolution volumetric SD-OCT dataset permits detailed examination of the trabecular meshwork (TM), Schlemm’s canal (SC) (red arrow) and the collector channels (CC) (white arrow). Images shown are obtained from dissecting the scanned tissue volume at a plane that gives optimal identification of collagen flaps to show the hinged flap or leaflet-like organization at the collector channel entrance. (a-f) Images are each oriented to provide an optimal view of collector channel relationships to SC, and the associated hinged collagen flaps (asterisks) that separate the collector channel from SC.). Each CC has a relatively long flap at its entrance creating the appearance of a hinged configuration. The hinged flaps or leaflets are each attached to the TM by means of thin cylindrical attachment structures (CAS) (black arrows) spanning SC. Some sections through the CAS revealed the presence of a lumen (green arrows).

With the 3D OCT dataset, the visualization plane can be manipulated, for example rotated, titled or shifted around each axis, to scrutinize microstructural details within the scanned tissue volume as seen in Fig 4. The figure presents a series of cross-sectional OCT images of the AOS structures obtained by examining a 3D OCT dataset at orientations chosen to optimally demonstrate the TM and SC as well as CCE in each cross-section. The optimal orientation was considered to be one that permitted identification of hinged collagen flaps (HCF) that are attached at one end to the external or corneoscleral wall of SC. The hinged configuration of the collagen flaps or leaflets provides the freedom to move around the plane of the hinge. Each of the HCF is linked to the TM by means of thin cylindrical attachment structures (CAS) (Fig 4). Some of the CAS has two walls with an intervening empty space that appears to be a lumen.

The current OCT setup has a field of view of 2x3 mm^2^, sufficiently large to visualize SC within a 2 mm segment of the limbal region. To provide an overall view of the radial SC lumen at controlled perfusion pressures, we scanned a series of adjacent OCT 3D scans. We then stitched the scans together to form a composite image to visualize SC morphology over a length of ~8 mm at a perfusion pressure of 50 mmHg. The result is shown in Fig 4a, demonstrating the presence of a cannula in one end of SC with the TM visible superior to SC.

**Fig 5:**
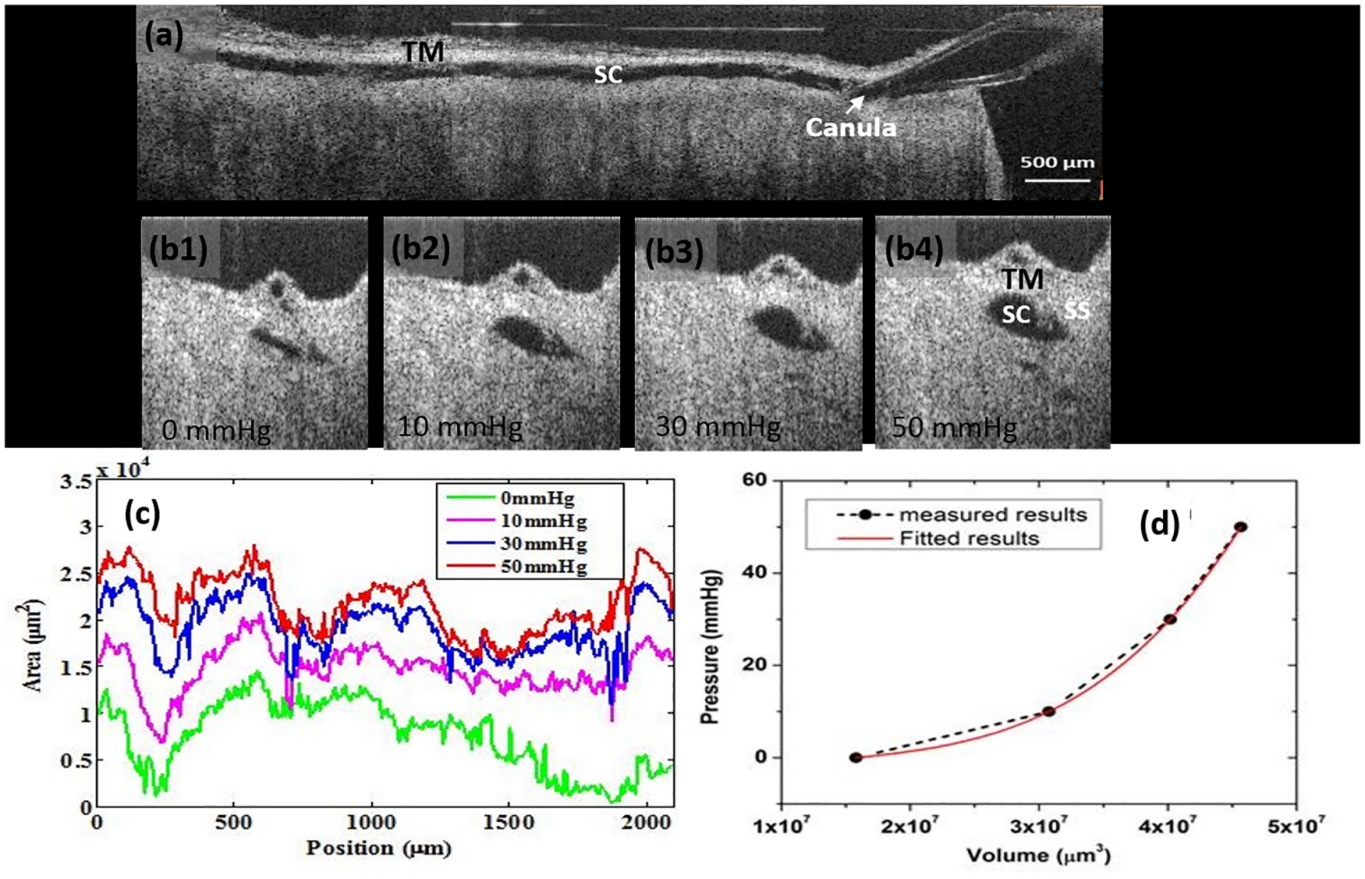
3D OCT imaging provides the ability to quantify the response of Schlemm’s canal to controlled perfusion pressures ranging from 0 of 50 mm Hg. (**a**) A composite cross-sectional image permits visualization of the entire length of ~8 mm of a limbal segment while maintaining a perfusion pressure of 50 mm Hg; SC is dilated along its entire length. A cannula is visible in SC at the right edge of the image of the segment and the trabecular meshwork (TM) is visible superior to the canal. OCT images (**b**1-**b**4) representing radial cross-sections through SC demonstrate progressive dilation of the canal as pressure increases from 0 to 50 mm Hg. (**c**) The curves represent the measured SC area at 10um intervals along the 2 mm limbal segment under pressures as indicated in the legend. (**d**) Elastance curve generated from measured SC volume increases resulting from changes in applied pressure.

Dilation of SC is maintained by the 50 mm Hg static pressure of the SC infusion cannula attached to the reservoir. Note that SC lumen remains dilated along its entire length. The degree of SC dilation differs at different pressures. Fig 5b shows representative OCT images scanned across the SC lumen under static pressure conditions of 0, 10, 30 and 50 mmHg, respectively; SC lumen progressively dilates with the increase of pressure. Changes in the sclera spur (SS) and TM configuration are also apparent.

The area along the longitudinal axis of SC dilated lumen can be quantified using the previously described automated segmentation algorithm [40]. Fig 5c shows the measurement curves along the 2 mm segment under different perfusion pressures; the volume of SC opening can be readily calculated from the curves. The calculations provide sufficient data to generate an elastance curve for the measured lumen volume; the result is shown in Fig 5d fitted to a polynomial curve.

It is known that in hollow tissues, as volume increases, part of the energy increases the pressure inside the tissue volume, while a portion of the energy alters the elastance [43]. Elastance is described as the tendency of a tissue surrounding a hollow organ to return to its original form after removal of a deforming force. Thus, we may explain Fig 5d as below. In the early part of the curve, as volume increases, much of the energy is used to impart elastic energy to the tissue so the pressure increases slowly. As volume continues to increase, the tissue capacity to absorb additional elastic energy progressively decreases causing the elastance curve to rise. A more rapid increase in pressure with further increases in volume is seen as tissue stiffens in association with movement along the elastance curve.

**Fig 6:**
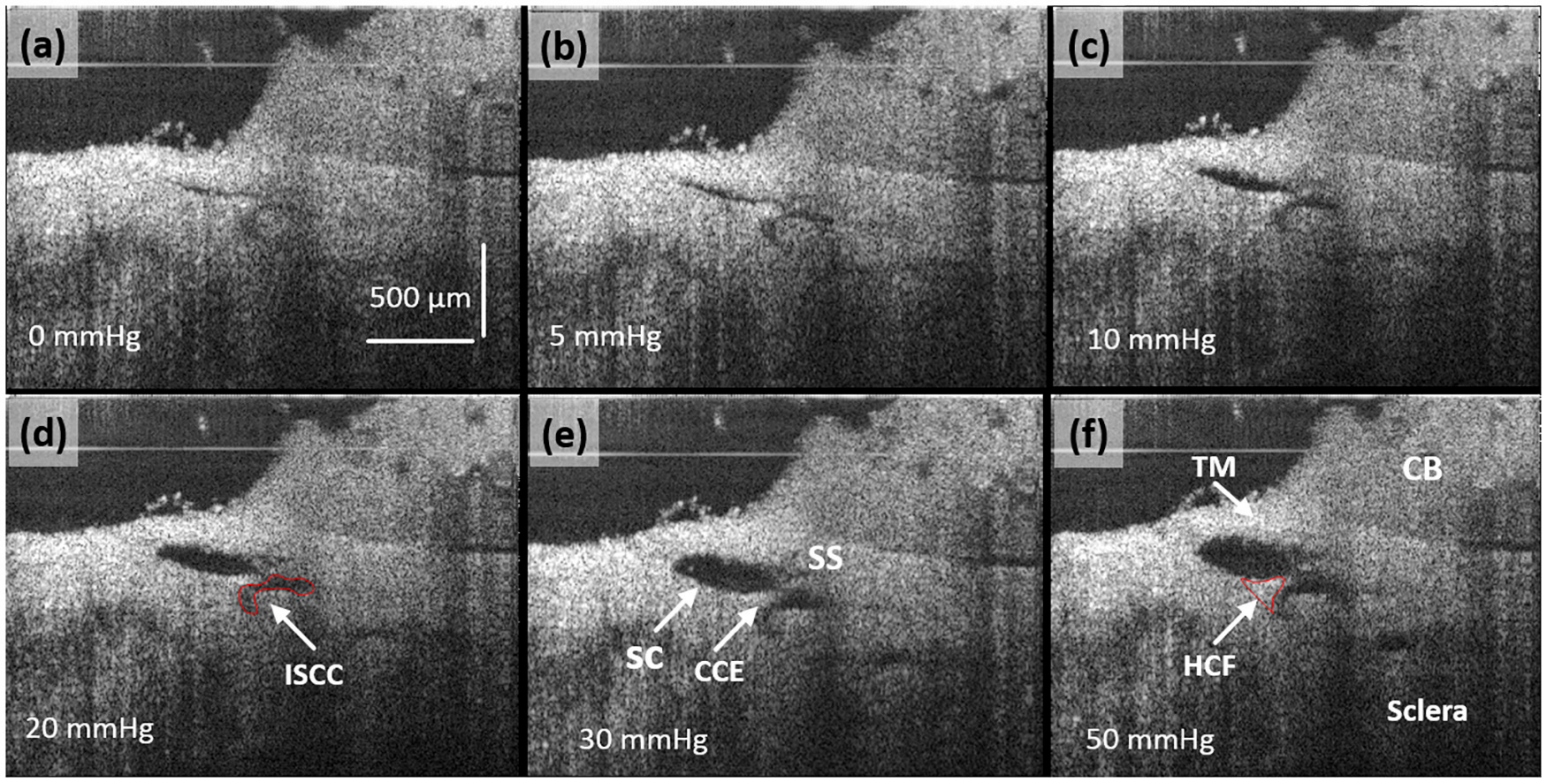
(a)–(f) OCT cross-sectional images under the steady state pressures of 0, 5, 10, 20, 30 and 50 mmHg, respectively. OCT imaging resolution is high enough to permit quantification of the configuration changes in Schlemm’s canal, collector channel entrances and intrascleral collector channels and their relationship under steady state pressure conditions. The ISCC in (d) and the HCF in (f) are outlined in red. Trabecular Meshwork (TM), Hinged Collagen Flap (HCF), Ciliary Body (CB).

OCT imaging provides an opportunity to examine changes in microstructures within the AOS in detail in response to the increase in lumen volume associated with the increases in reservoir pressures. In these experiments, steady state pressure was achieved by maintaining known reservoir heights (Fig 1). Fig 6 demonstrates easily recognizable increases in SC, CCE and ISCC lumen dimensions when the pressure was increased from 0 through 5, 10, 20, 30, to 50 mmHg. At 0 mmHg, the TM is visible, but SC, the CCE and the ISCC appear to be little more than a potential space. At 50 mmHg, the individual tissue compartments become apparent in the OCT image. The SS and ciliary body (CB) are attached to the TM. A HCF is visible on each side of the CCE. With the increase of pressure the lumens of SC, CCE, and ISCC progressively enlarge. The angle of the SS relative to the sclera also moves superiorly in the image. The CCE becomes more easily visible. A thin structure is visible spanning between the TM and the HCF closest to the SS. A HCF at the CCE also appears to change its relationships to the surrounding structures.

**Fig 7:**
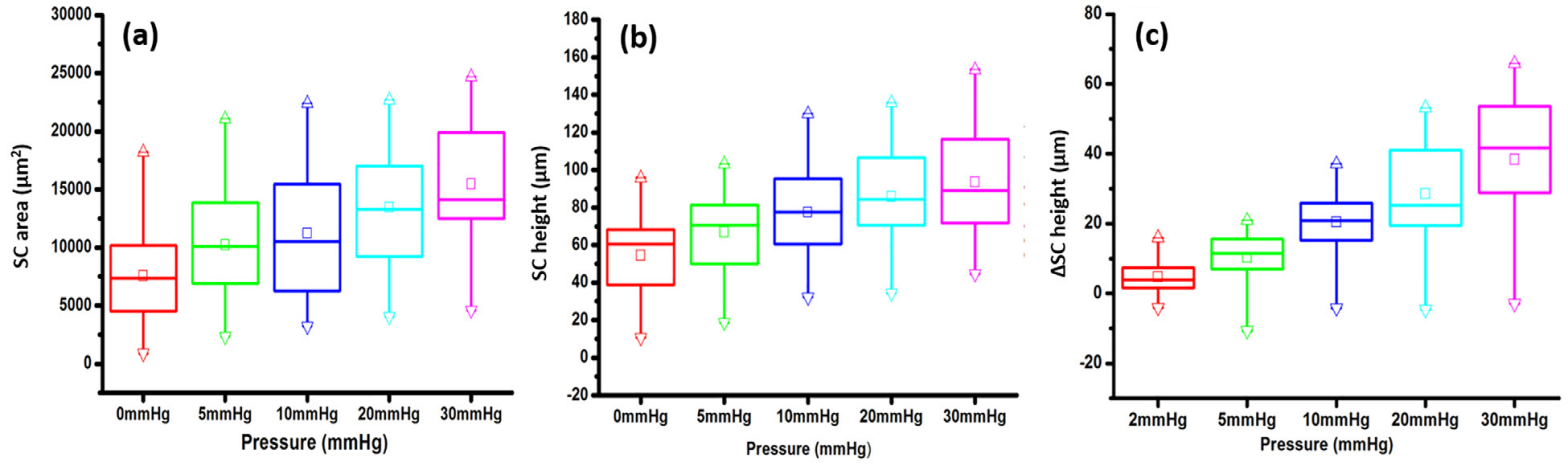
(A), (B), and (C) are boxplots of the data for Schlemm’s canal (SC) area, height and height change respectively. Data is from 14 quadrants of 4 eyes in A & B and from 13 quadrants in C. SC height changes in C are reflected in the data of Fig 7, which displays normalized differences of individual quadrants. The boxplot central lines represent the median, and the box borders the interquartile distance. Means are represented by the small squares. Maximum value are shown as Δ, while minimum as inverted Δ. *p<0.05. The whiskers represent the 99% and 1% range of Tukey.

In the 14 quadrants of the 4 eyes, SC height and area data are summarized in the box plots of Fig 7a and 7b respectively. Individual quadrants have marked dimension variability as is illustrated by the relatively wide range of interquartile values. In one of the SC areas and in 3 of the SC height boxplots the data is not symmetrically distributed. However, both the area and height data exhibit a progressive increase in mean, median and interquartile values as perfusion pressure increases.

The change in SC height in response to changes in pressure is depicted in Fig 7c where a progressive increase in mean, median and interquartile values is seen. There is marked variability from quadrant to quadrant as is illustrated by the 1 and 99^th^ percentile whisker values. Comparison of pressure-dependent height changes in the 14 individual quadrants provides a more complete picture because it eliminates the confounding variable of the marked variability between individual segments (Table A in S1 File). The SC area, height and height change were evaluated by ANOVA. Twelve of 14 values reached significance at the 0.01 level. Polynomial fitting of the curves for the 14 quadrants provided an *r*^*2*^ value of > 0.80 in all quadrants for area, as well as for 11 quadrants for height and 11 quadrants for height change. The corresponding *p*-values for *r*^*2*^ were <0.001 in all quadrants for area, 11 quadrants for height and 11 quadrants for height change. SC height responses do not follow a predictable pattern in individual eyes or individual quadrants.

Tukey paired comparisons for SC area for each eye demonstrate p values of <0.05 for 0–20, 0–30; 5–20, 5–30; and 10–30 mmHg pressure differences respectively. Tukey paired comparisons for SC height demonstrate p values <0.05 for 0–10, 0–20, 0–30; 5–20, 5–30; and also10-30 mmHg pressure differences respectively. Tukey paired comparisons for SC height demonstrate p values <0.05 for 5–10, 5–20, 5–30; 10–20, 10–30 and 20–30 mmHg pressure differences respectively.

**Fig 8:**
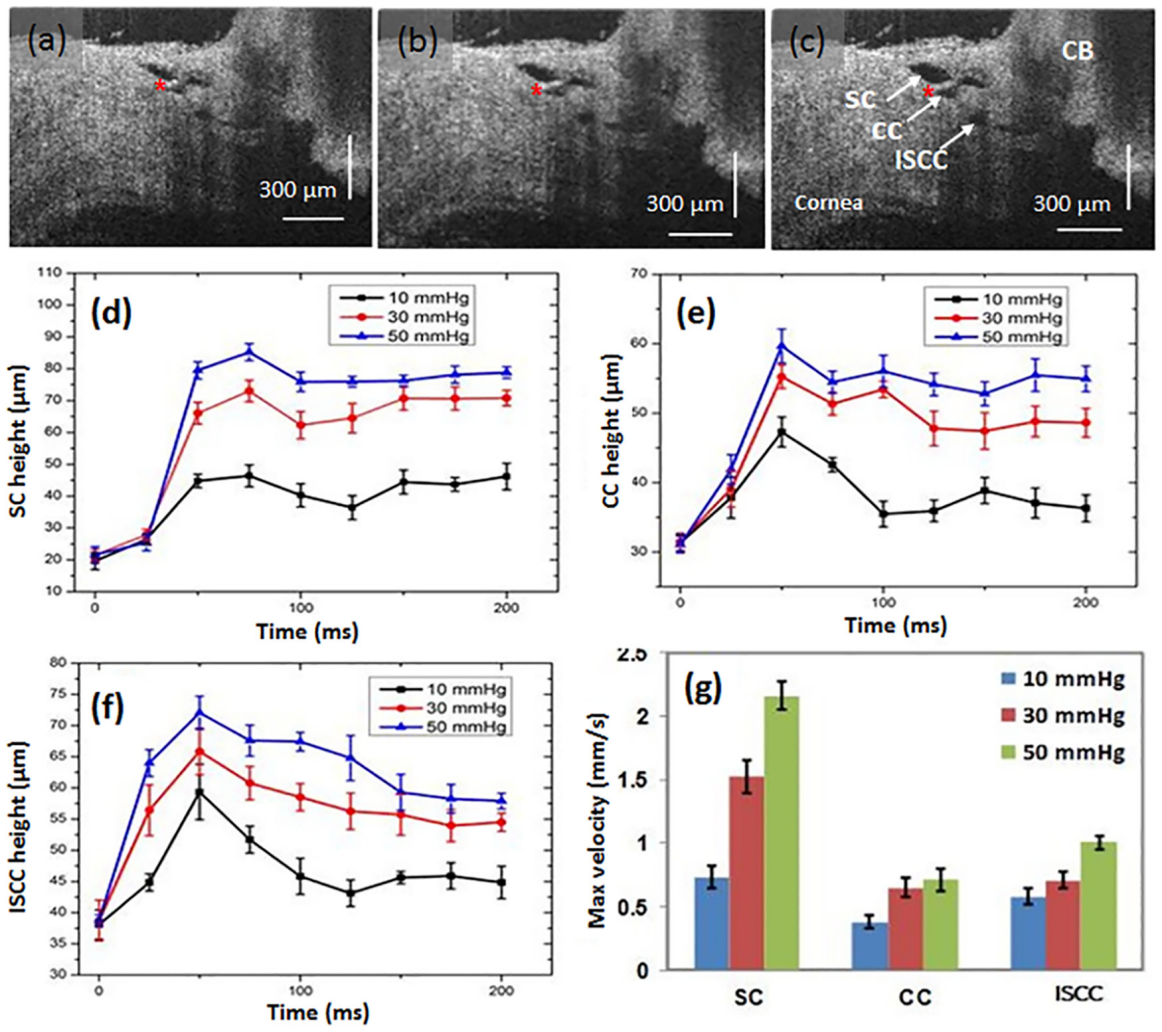
OCT images (a-c) represent a section through the limbus while maintaining cannula pressures of 10, 30, and 50 mm Hg respectively; the images represent the static configuration after having switched to the respective reservoir pressures from a baseline of 0 mm Hg pressure. The ciliary body (CB), trabecular meshwork (TM), Schlemm’s canal (SC), collector channel (CC) and intrascleral collector channels (ISCC) undergo progressive changes in shape in response to pressure increases. Red asterisks indicate the base of a hinged collagen flap. Height changes with time of the lumen of SC, CC and ISCC are depicted in (d) (e) and (f); time was determined from the initiation of height change following switching from a baseline reservoir height of 0 to a height of 10, 30 or 50 mm Hg respectively. The bar chart (g) depicts maximum velocities of SC, CC and ISCC lumen height change following pressure changes from the 0 baseline to the10, 30, or 50 mm Hg reservoir height.

Fig 8 illustrates time-dependent changes in SC, CC and ISCC lumen heights. The images are from discrete SD-OCT video frames. The first frame where movement was detected was identified and the frame prior to that was used as the reference frame. Although amplitudes differ, the changes in lumen height appear to be synchronous. Maximum increases in lumen height occur within about 50 msec at each of the pressures, followed by a subsequent plateau or slight decrease in height for all the measurements. The later height decrease after the peak is particularly apparent at 10 mm Hg in the CC and ISCC lumen areas. The later decrease may result because compared with SC lumen, the CC and ISCC lumen are both more distant from the cannula and also closer to the intrascleral channels that discharge aqueous. The maximum velocity of the change in lumen wall dimensions is greatest in SC compared with the more distal pathway CC and ISCC lumen dimensions changes.

**Fig 9:**
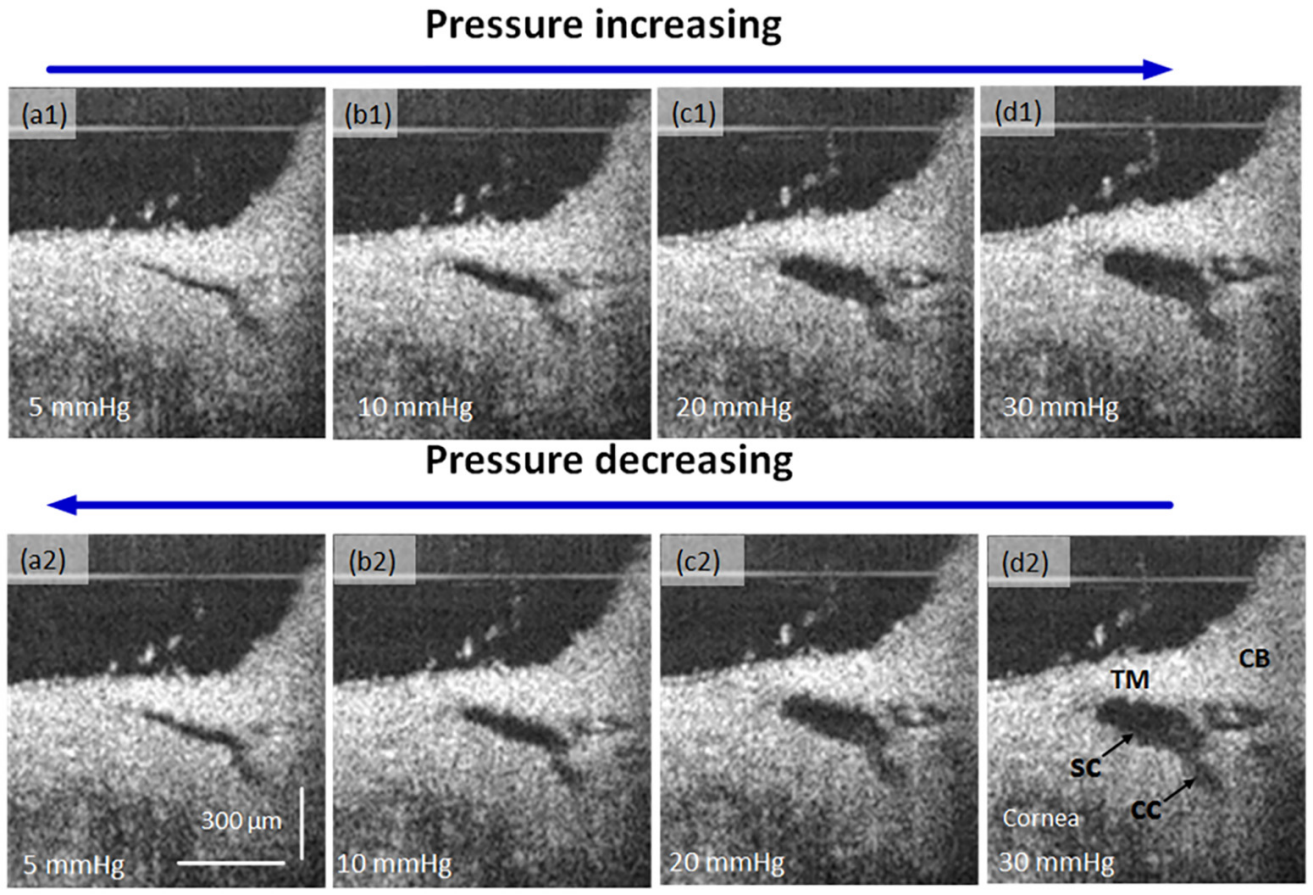
OCT cross-sectional images sequentially acquired at static progressively increasing perfusion pressures of 5, 10, 20 and 30 mmHg (top row) and then sequential acquisition in the reverse direction (bottom row). Ciliary Body (CB), TM Trabecular Meshwork (TM), Schlemm’s canal (SC), Collector Channel (CC).

To examine the elastic properties of the AOS, OCT images were acquired sequentially by the increase of static perfusion pressures of 5, 10, 20, and 30 mmHg. Stepwise sequential reduction of the perfusion pressure to 5 mmHg follows the previous acquisition at increasing pressures. Fig 9 presents the stepwise perfusion results. Gross examination of images at 5 and 10 mm Hg reveal a larger SC and CC cross-sectional area in the decreasing loop of the pressure curve than that in the increasing loop at the same pressure. The difference is less apparent at 20 and 30 mm Hg.

## Discussion

The current study demonstrates pressure-dependent AOS tissue relationships in *ex vivo* human eyes using our recently developed high-resolution OCT platform. We describe evidence of TM, SC, and CCE tissue displacement responses, their linked lumen dimension responses and the synchrony of this pressure-dependent behavior. A major strength of the current study is its ability to simulate clinical studies that identify abnormal outflow system responses; such responses in glaucoma are thought to be consistent with tissue stiffening [1–7]. Abnormal stiffening is thought to result from alterations in biomechanical properties of the AOS tissues [15]. In fact, studies have shown about a 20-fold increase in tissue stiffness in glaucoma compared that in normal [13]. Although both cellular and extracellular constituents of the AOS may play important roles, tissue mechanics remain an underexplored component of the glaucoma processes associated with IOP elevation [15].

Studies of cytoskeletal mechanics [44,45] as well as study of morphology and constituent properties [46] are consistent with the conclusion that altered AOS tissue stiffness is involved in the abnormal regulation of aqueous flow in glaucoma. Systemic markers for ECM alterations have been shown to be present in glaucoma patients [47–51]. Biophysical signaling within the TM is important to TM cell function but such cues are still not well understood [52–56].

Some clues include evidence that steroid-induced glaucoma is associated with the increase of deposition of ECM proteins in trabecular beams, in the uveal meshwork and in the JCT region [46,57,58]. Recently, dexamethasone-induced biomechanical changes in the TM cells, ECM and tissue have been linked to outflow pathway abnormalities both *ex vivo* and *in vivo*. Such changes suggest steroid-induced pressure-elevation results from stiffening of TM tissue [15].

Clinical correlates to the laboratory evidence of AOS tissue stiffening can be found in many studies of pulsatile flow from SC into aqueous veins. Pulsatile flow from SC into the aqueous veins in synchrony with the ocular pulse can be best explained by trabecular tissue motion altering SC dimensions. Such pulse-induced TM motion has recently been demonstrated with OCT in both *ex vivo* and *in vivo* studies [30–32].

Slit-lamp studies demonstrate pulsatile flow abnormalities in glaucoma that are thought to result from abnormal AOS tissue motion. Progressive reduction in pulsatile flow occurs as glaucoma worsens, likely resulting from gradual loss of TM elasticity and compliance [59–62]. Medications that reduce IOP in glaucoma restore pulsatile aqueous outflow, indicative of the ability to improve TM motion [59,62,63].

The goal of biomechanics is to study tissue behavior that is applicable to processes that occur in vivo both in health and in disease. We feel our experimental design is especially strong because it fulfills the goal of mirroring behavior that occurs under both physiologic and pathologic conditions in vivo. We also replicate EVP pressure reversal used for in vivo studies that has been so effective in demonstrating outflow abnormalities in glaucoma. Syndromes that cause chronic SC pressure reversal also cause intractable glaucoma further emphasizing the need to replicate in vivo conditions [64, 65]. Following inversion, studies report an IOP increase within seconds from the mid-teens to mid-30s that occurs as a result of EVP elevation. IOP and SC pressures rise to as high as 43 mm Hg following such inversions with associated SC filling with blood [66–68]. Knowledge of these physiologic responses is what led us to bracket SC pressure readings between the ranges of 0 and 50 mm Hg.

Pressure reversal that causes IOP to be lower than EVP is achieved by several readily available clinical gonioscopy techniques. Normally, the SC lumen is little more than a potential space. However, in normal subjects gonioscopy studies have shown that a reversal of pressure relationships results in IOP lower than EVP and causes blood to rapidly dilate and fill the lumen of SC [1,5,69,70]. Dilation of SC results from the TM rapidly collapsing toward the AC, increasing the dimensions of the SC lumen [8,9].

These same studies have also demonstrated that in normal subjects with restoration of normal gradients, (IOP > EVP), blood rapidly leaves the lumen of SC. The filling and emptying phenomena are dependent upon the intrinsic compliance and elasticity of the TM tissues. In mild glaucoma, blood fills and empties from SC slowly. In more advanced glaucoma, there is only patchy filling of the canal with blood and in far advanced glaucoma SC filling with pressure reversal is absent [1,5,69,70].

These clinical studies provide physiologic evidence of a progressive pathologic process in glaucoma that involves alterations in biomechanical properties of both the TM and distal outflow system. Recent studies involving minimally invasive glaucoma surgery take advantage of this knowledge to provide a predictive tool for the success or failure of a non-penetrating surgery referred to as canaloplasty [69].

Our current study is an effort to mirror these clinical techniques that have been so valuable in exploring pathologic mechanisms in glaucoma. Our ability to image and quantitate both static and dynamic processes provides a means to study normal AOS tissue properties. Such studies can then be used for comparison with the changes of these tissue properties in glaucoma eyes. Alterations in biomechanical properties of the AOS are currently the focus of intense study in the laboratory [14,15,71]. The high-resolution characteristics of our OCT platform provide the ability to develop 3D projections to explore not only SC, but also CC and ISCC relationships. Using this platform in conjunction with 3D visualization software permits the selection of the optimal view to examine the configuration and dimensions of the lumen of CCE in 3D space.

The collector channel entrances have unique features initially described by Rohen as lip-like thickenings of the external wall with short oblique septa [42]. He demonstrated their presence and the attachments to SC inner wall through use of serial histologic sections. He recognized these collector channel entrance structures could close and pointed out that they are likely held open because of their attachment to SC inner wall.

Recently, three-dimensional micro-computed tomography (3D micro-CT) studies further demonstrated that collector channel entrances change shape with immersion fixed eyes having a mean orifice size of 27.5±5 μm while eyes fixed at 10 mm Hg IOP had a mean orifice size of 40.5±13 μm, indicating the ability of collector channel ostia to move, a behavior that would not be expected if they were simply encased in a rigid collagen entrance [72].

Another 3D micro-CT study demonstrated variable collector channel entrance occlusions in normal and glaucoma eyes with a 3-fold increase in total occlusions of collector channel ostia in glaucoma eyes. Visualization of collector channels increased by 24% in normal and by 21% in glaucoma eyes at 20 mm Hg compared with 10 mm Hg, further demonstrating the presence of tissue geometry that permits collector channel entrance motion [73]. Studies by the same group using scanning electron microscopy (SEM) provides 3-D evidence of typical flap-like structures that extend over the collector channel orifices and also attach to the SC inner wall [74].

An additional study uses SEM to visualize 3D evidence of hinged collagen flaps at CCE and compares them with high-resolution images obtained by OCT which also demonstrates pressure-dependent collector channel motion [29]. Pressure-dependent motion of the hinged collagen flaps been demonstrated through use of serial histologic sections. These same serial histologic sections demonstrated the transition from the hinged area to the intrasceral channels [75].

The current high resolution OCT study provides further evidence of the presence of regularly recurring flaps or leaflets at the CCE. The flaps anchor to the external or scleral wall at only one end creating a hinge. The hinged configuration permits the flaps to move around the plane of the hinge. The 3D OCT imaging also permits identification of regularly recurring cylindrical attachments that span across SC to connect the hinged CC leaflets to the TM, a relationship originally recognized by Rohen [42].

High-resolution reconstruction of a long region of SC in conjunction with development of an algorithm to segment SC lumen permits establishment of an SC volume profile at different pressures. It is then possible to generate an SC elastance curve based on the volume related pressure changes. Under our experimental conditions, SC acts as a 3-dimensional chamber or reservoir, a fluid containing hollow organ. The mechanical properties of hollow organs are described by their elastance, a parameter widely used in cardiovascular and pulmonary physiology [76,77].

Elastance is described as the tendency of a hollow organ to return to its previous dimensions upon removal of a distending force [78,79]. Young’s modulus is described as the tendency of an object to distend along its axis, whereas elastance is a bulk modulus that can be thought of as Young’s modulus in 3 dimensions. Each parameter is reflective of the tissue stiffness, a property recently recognized as likely to be of major importance in the understanding of the glaucoma process [15,52].

Marked variability between quadrants in individual eyes is identified when our study measures the static pressure-dependent configuration, a finding probably reflective of regional differences in tissue configuration. However, pooled data from quadrants of the 4 eyes showed a pressure-dependent increase in SC area and height as well as the changes in the SC height. Tukey paired comparisons demonstrated a lack of significant configuration change between 0 and 5 mm Hg values, but comparisons involving higher pressure differences are significant. As pressure in SC increased from 0 to 5 mm Hg, the TM tissues are subjected to a normal stress or load that was previously absent. A pre-stress or preload identified by pressure-dependent TM distention from loading pressures is present *in vivo* and may better reflect normal tissue behavior [8,9,11].

When we measure dynamic pressure-dependent changes, we find motion of the TM, CCEs and ISCC that is synchronous. The presence of synchronous motion is not surprising since the HCF at CCEs are attached to the TM via the cylindrical structures spanning SC in a tensionally integrated relationship [12,29,42,80]. The presence of the TM-HCF attachments suggests that the well-documented pressure-dependent changes of the TM will necessarily require pressure-dependent changes of the HCF at CCE. The linkage suggests the hypothesis that the TM-HCF linkage provides a means for pressure to control the CCE configuration.

Limitations to be considered in our current study include the lack of ciliary body tension, as well as a lack of normal EVP, issues inherent to all *ex vivo* studies. An additional limitation is the possibility that flow from SC backward into the AC would confound the results. However, both *ex vivo* [8,9,81,82] and *in vivo* [5,65,69,70] studies show that blood, and hence aqueous, does not flow from SC into the AC because the TM acts as a barrier to retrograde flow. The SC pressure-gradient reversal in this study leads to a configuration that may occur transiently under physiologic conditions. Transient pressure reversal in SC is well recognized as a physiologic phenomenon in the context of various fitness activities, which at times include body inversions. Following inversions, marked pressure rises and entry of blood into SC occurs within seconds [66– 68].

Tissue viability is a possible concern. In this study, all tissues were received and experiments initiated within an interval of less than 24 hours after death. Studies demonstrate that corneoscleral explants containing the outflow system can be maintained for several weeks postmortem and only begin to display minor cellular and extracellular matrix degradation after 3 weeks in culture [83]. Additional studies demonstrate live cell viability [84] and ability to view actin polymerization as long as 6–7 days post mortem [85]. Our studies were done in tissues <24 hours post mortem suggesting that our system could be of useful to evaluate the effects of pharmacologic agents on SC morphology and deformability.

Because we are using only segments from eyes, rapid flow out of the cut end of SC might occur; CCE along SC may further divert the flow as fluid progressively flows along SC. These confounding factors prevent us from knowing the exact pressure within SC at any individual location.

These potentially confounding variables can be addressed to some extent by examining our experimental data in more detail. The dimensions of SC, CCE and ISCC are small in relation to the fluid volume introduced into the lumen of SC in our technique. Findings from our study as well as the previous study in non-human primate eyes [29] suggest that pressure in the canal may remain relatively stable for two reasons. First, the canal is dilated not only in the proximal portion, but also distally far from the cannula tip. The consistent dilation along the length of the canal suggests that pressure remains relatively stable throughout SC length. Second, the graded pressure-dependent responses of the TM tissue at locations far from the cannula further suggest that a steady state pressure is present in the canal. Our experimental results also indicate that within the time interval from beginning to end at each of the pressures applied, the SC volume stays quite stable (with an average variation of <2%) at positions both close to the cannula and closer to the cut ends of SC (Fig A in S1 File). Therefore, we conclude that the SC pressure remains stable when set to specific steady state levels.

The current paper provides evidence that in human eyes the TM is in motion and responds to small pressure gradient changes in milliseconds. The results additionally provide evidence that hinged collagen flaps or leaflets are present at the CCE. The CCE leaflets are linked to the TM and the linked structures undergo synchronous motion in response to the changes in pressure. An estimate of elastance, a stiffness parameter can be developed from measurements of pressure-dependent AOS dimension changes.

## Summary

We have demonstrated a high-resolution OCT platform capable of imaging the AOS of human eyes from the trabecular meshwork surface, providing details of the TM, SC, CCE and their synchronous pressure-dependent motion. We found evidence that the tissues surrounding the lumen of SC and the CC respond to the changes in pressure within milliseconds. Evidence from clinical and laboratory studies suggest that the ability to move is diminished and eventually lost in glaucoma. Advances in OCT imaging may provide new clinical insights into the mechanism and time course of the decrease in AOS tissue motion in glaucoma. Such insights derived from rapidly evolving OCT imaging technologies may lead to clinical tools that can provide improved guidance for management of glaucoma.

## Supporting Information

S1 File Supplementary Information. **Table A:** Schlemm’s canal area, height and height change in each quadrant of the 4 eyes evaluated by Anova and polynomial fitting. Inferior Nasal (IN), Inferior Temporal (IT), Superior Temporal (ST), Superior Nasal (SN). **Fig A:** The SC pressure remains stable when set to specific steady state levels. The experimental results indicate that within the time interval from beginning to end at each of the pressures applied, the SC volume stays quite stable (with an average variation of <2%) at positions both close to the cannula and closer to the cut ends of SC.
(DOCX)
