# Supplementary material for: OCT Study of Mechanical Properties Associated with Trabecular Meshwork and Collector Channel Motion in Human Eyes
Source: PLoS One. 2016 Sep 6;11(9):e0162048. doi: 10.1371/journal.pone.0162048 (PMC5012558; doi:10.1371/journal.pone.0162048)
Supplement: S1 File — Table A: Schlemm’s canal area, height and height change in each quadrant of the 4 eyes evaluated by Anova and polynomial fitting. Inferior Nasal (IN), Inferior Temporal (IT), Superior Temporal (ST), Superior Nasal (SN). Fig A: The SC pressure remains stable when set to specific steady state levels. The experimental results indicate that within the time interval from beginning to end at each of the pressures applied, the SC volume stays quite stable (with an average variation of <2%) at positions both close to the cannula and closer to the cut ends of SC. (DOCX) [file pone.0162048.s001.docx]

Supplementary Information

**Table A**

Schlemm’s canal area, height and height change in each quadrant of the 4 eyes evaluated by Anova and polynomial fitting. Inferior Nasal (IN), Inferior Temporal (IT), Superior Temporal (ST), Superior Nasal (SN).


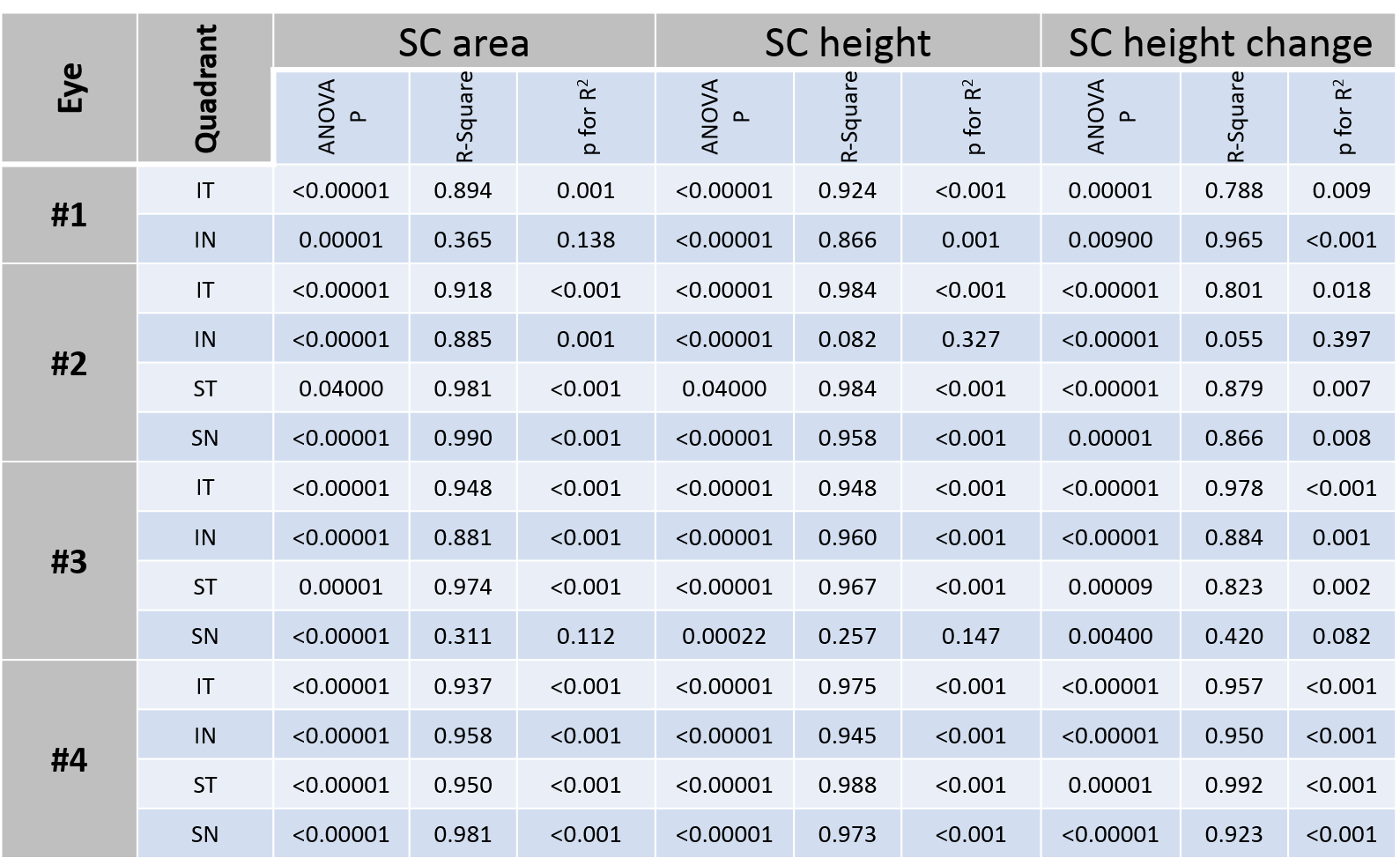


**Figure A**

The SC pressure remains stable when set to specific steady state levels. The experimental results indicate that within the time interval from beginning to end at each of the pressures applied, the SC volume stays quite stable (with an average variation of <2%) at positions both close to the cannula and closer to the cut ends of SC.


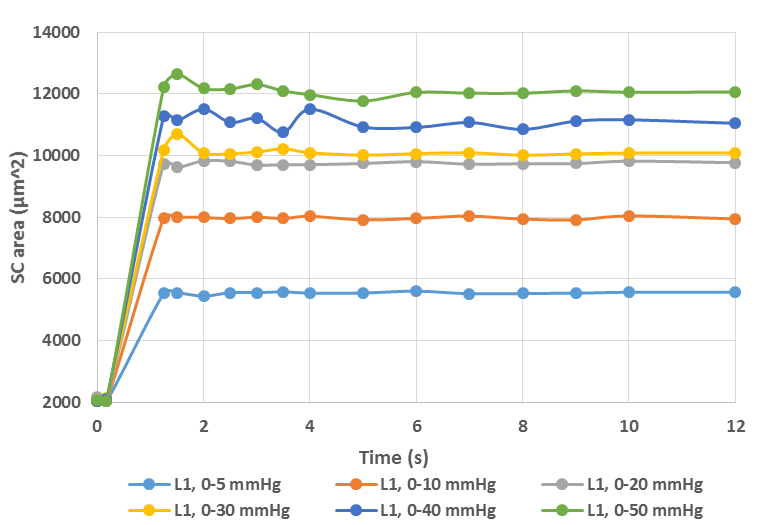


SC volume changes over time when the SC pressure set to specific pressure levels, measured at location close to the cannula.


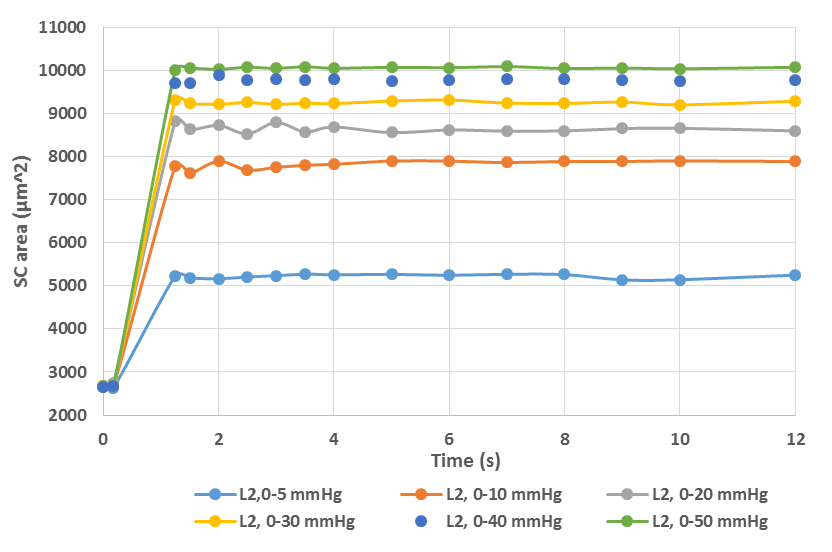


SC volume changes over time when the SC pressure set to specific pressure levels, measured at location close to the cut ends of SC.
